# Golgi protein 73 activation of MMP-13 promotes hepatocellular carcinoma cell invasion

**DOI:** 10.18632/oncotarget.5590

**Published:** 2015-09-10

**Authors:** Di Jin, Jun Tao, Dan Li, Yanan Wang, Li Li, Zhongdong Hu, Zhenzhen Zhou, Xiuli Chang, Chunfeng Qu, Hongbing Zhang

**Affiliations:** ^1^ Institute of Cancer Stem Cell, Dalian Medical University, Dalian, China; ^2^ State Key Laboratory of Medical Molecular Biology, Department of Physiology, Institute of Basic Medical Sciences, Chinese Academy of Medical Sciences and Peking Union Medical College, Beijing, China; ^3^ Cell Engineering Research Center, Chinese Academy of Medical Sciences and Peking Union Medical College, Beijing, China; ^4^ Modern Research Center for Traditional Chinese Medicine, Beijing University of Chinese Medicine, Beijing, China; ^5^ Department of Physiology, Dalian Medical University, Dalian, China; ^6^ State Key Laboratory of Molecular Oncology, Cancer Institute/Hospital, Chinese Academy of Medical Sciences, Beijing, China

**Keywords:** GP73, matrix metalloproteinase-13, hepatocellular carcinoma, invasion

## Abstract

Golgi Protein 73 (GP73) is a serum biomarker for hepatocellular carcinoma (HCC), however its role in HCC is not clear. We report that GP73 promotes cell invasion, the hallmark of malignancy, through the upregulation of matrix metalloproteinase-13 (MMP-13). GP73 enhances MMP-13 expression through cAMP responsive element binding protein (CREB)-mediated transcription activation. Levels of GP73 and MMP-13 are increased and positively correlated in human HCC tissues. Augmented MMP-13 potentiates HCC cell metastasis. Thus, the GP73-CREB-MMP-13 axis potentiates cancer cell invasion and may be a target for HCC treatment.

## INTRODUCTION

Hepatocellular carcinoma (HCC) is the fifth-most common cancer worldwide and the third-leading cause of cancer death [1]. The high mortality rate of HCC is mainly attributable to a late diagnosis at an advanced stage. Even though alpha-fetoprotein (AFP) is a specific serum marker for HCC, it is of minimal use as a diagnostic tool because its sensitivity is only around 50% [2]. Among other reported markers, Golgi protein 73 (GP73), also named Golgi membrane protein 1 (Golm1) or Golgi phos­phoprotein 2 (GOLPH2), is proving to be a better biomarker for HCC [3–5].

GP73 is a 73-kDa type-II Golgi transmembrane glycoprotein that was originally cloned from a library derived from the liver tissue of a patient with adult giant-cell hepatitis [6]. Knowledge on the structure and function of GP73 is limited. Aberrant over-expression of GP73 has been reported to correlate with many diseases such as viral infections, Alzheimer's disease, Wilson's disease and cancer [6–10]. Despite its steady-state localization in the Golgi ap­paratus, GP73 can be secreted into the extracelluar space by cleavage at a proprotein convertase (PC) site and is present in the culture media of sev­eral cell lines [11, 12]. Block and colleagues reported that serum GP73 (sGP73) levels were up-regulated in patients with hepatitis B virus-related HCC [12]. We, and others, demonstrate that the sensitivity and specificity of sGP73 for the identification of HCC are superior to those of alpha-fetoprotein (AFP), especially in early HCC [4, 13]. Thus, we propose sGP73 as a novel marker for HCC diagnosis.

The discovery of GP73 as a potential biomarker for hepatocellular cancer begs the question as to its relevance in human HCC development. GP73 is a contributor to fibrogenesis in patients with chronic HBV infections, and GP73 recombinant protein can prompt the proliferation of LX2 cells (hepatic stellate cell line) [14]. Clinical results from several groups showed that an elevated level of GP73 protein is strongly associated with tumor size, vein invasion, tumor differentiation, epithelial–mesenchymal transition and overall survival [15–17]. Recently, we found that GP73 promotes cell proliferation, migration, invasion and metastasis [18]. Our studies suggest that GP73 plays an important role in tumor growth and metastasis. Nevertheless, the underlying mechanism is largely unknown.

In this study, we found that GP73 potentiated MMP-13 expression and that both GP73 and MMP-13 were higher in human HCC tissues compared to adjacent liver tissues. We then identified cAMP responsive element binding protein (CREB) as the mechanistic link between GP73 and MMP-13. GP73 increased CREB expression and then the enhanced CREB transcriptionally activated MMP-13. This GP73-CREB-MMP-13 signaling pathway was able to promote HCC cell invasion. Lastly, augmented MMP-13 potentiated HCC cell metastasis in nude mice.

## RESULTS

### GP73 potentiates MMP-13 expression in HCC cells

**Figure 1:**
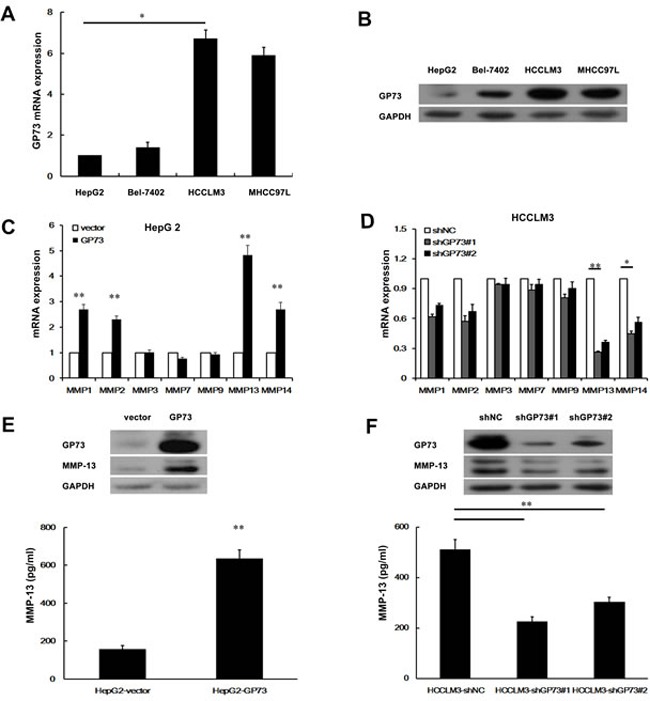
GP73 enhances MMP-13 expression Relative levels of GP73 in 4 liver cancer cell lines were detected by qRT-PCR **A.** and Western blot **B.**. qRT-PCR analysis of MMPs mRNA level in HepG2 cells with GP73 overexpression **C.** or HCCLM3 cells with GP73 knockdown **D.**. Cellular levels of GP73 and MMP-13 in HepG2 cells with GP73 overexpression (**E.**, top) or HCCLM3 cells with GP73 knockdown (**F.**, top). The levels of secreted MMP-13 (active form) in the culture supernatants were measured by ELISA (**E.**, **F.**, bottom). A *t*-test was used to evaluate the statistical significance of these experiments, as compared to the control. *P < 0.05; **P < 0.01.

**Figure 2:**
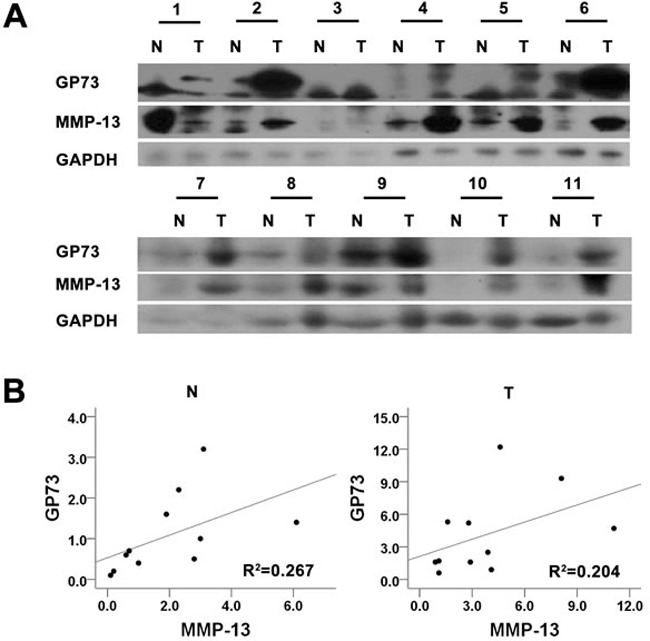
GP73 and MMP-13 are up-regulated in HCC tissues Tumors and the adjacent liver tissues from 11 HCC patients were prepared for Western blot analysis of GP73 and MMP-13 expression **A.**. The abundance of GP73 and MMP-13 was then analyzed **B.**.

We first used quantitative real-time PCR (qRT-PCR) and Western blots to examine GP73 expression in 4 human liver cancer cell lines that had different metastatic potentials (Figure 1A, 1B). Higher GP73 expression was observed in the more invasive cell lines, such as HCCLM3 and MHCC97L, than in HepG2 and Bel-7402. We therefore selected a higher GP73 expression cell line, HCCLM3, and a lower one, HepG2, for further investigation. To determine how GP73 facilitates HCC cell invasion, we checked for the expression of several proteolytic enzymes involved in degrading basement membranes. These included 7 matrix metalloproteinases (MMPs) that we identified by qRT-PCR analysis and Western blot in HepG2 cells with ectopic GP73 expression and in HCCLM3 cells depleted of GP73. We found that 4 MMPs, MMP-1, MMP-2, MMP-13 and MMP-14, were increased after ectopic GP73 expression (Figure 1C) and decreased after depletion of GP73 expression. Three other MMPS, MMP-3, MMP-7, and MMP-9, were not affected by GP73 state (Figure 1D). Since alteration of MMP-13 was most prominent among the first 4 MMPs, we confirmed the ability of GP73 to increase MMP-13 levels with Western blot of cell lysates and ELISA of conditioned media (Figure 1E–1F). To determine the potential correlation of GP73 and MMP-13 in HCC tissues, we checked the expression of GP73 and MMP-13 in primary tumors from HCC patients with Western blot (Figure 2A) (Table 1). In comparison with adjacent normal liver tissues, both GP73 and MMP-13 were both overexpressed in most of the tumors we checked (Figure 2B).

**Table 1:** Clinicopathologic characteristics of patients with hepatocellular carcinoma

No.	Age	Differentiation	Microvascular invasion	Cirrhosis	HBV	HCV	AFP (ng/ml)
1	57	Low	−	+	+	−	27
2	55	Low	−	+	+	−	21.28
3	74	Low	−	+	+	−	2
4	64	Low	−	+	+	−	83
5	64	Low	−	+	+	−	6.92
6	68	Low	+	+	+	+	2.7
7	47	High	−	+	+	−	1.73
8	74	High	−	−	+	−	1.25
9	27	High	−	+	+	−	2
10	58	High	−	+	+	−	2.7
11	31	High	−	−	+	−	417.8

### Overexpression of MMP-13 promotes HCC cell invasion and metastasis

**Figure 3:**
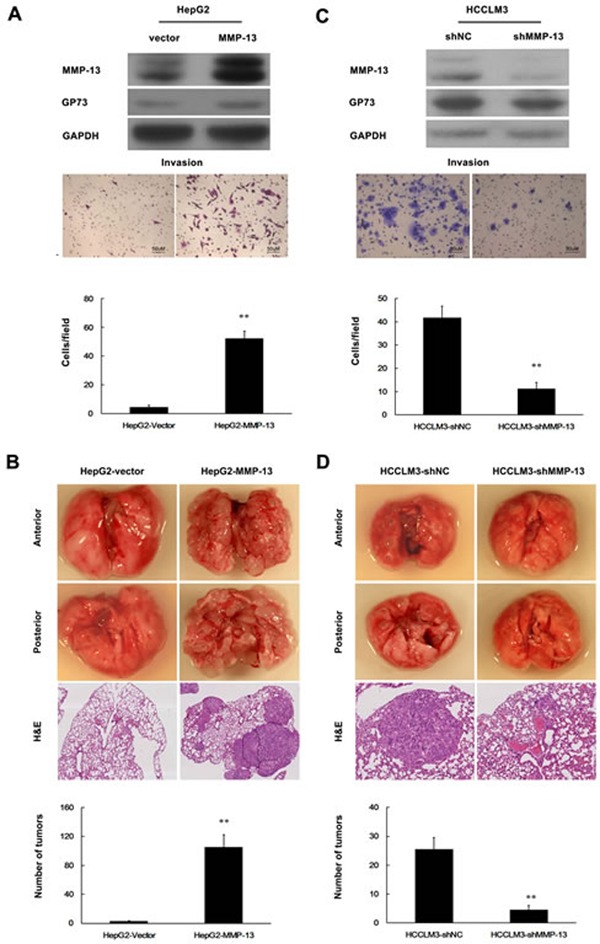
MMP-13 enhances invasion and metastasis of HCC cells **A.**, **C.** Cell invasion was assessed by Matrigel Transwell assay. **A.** HepG2 cells were stably transfected with PCDNA6-MMP-13 (HepG2-MMP-13) or PCDNA6 (HepG2-Vector; control). **C.** MMP-13 was knocked down in HCCLM3 cells. Western blot (top), transferred cells (magnification, 200×) (middle) and the histograms of transferred cells from triplicate tests (mean ±SD) (bottom). **B.**, **D.** Tail vein injection of cells was used for lung metastasis. **B.** Ectopic MMP-13 was stably expressed in HepG2 cells. **D.** MMP-13 was knocked down in HCCLM3 cells. Representative lung metastases (top), H&E staining of the lung tissues (middle) and scattergram of the numbers of tumor nodules in 4 nude mice during 10 weeks of observation (bottom). A *t*-test was used to evaluate the statistical significance of these experiments, as compared to the control. ** *P* < 0.01.

Upregulation of MMP-13 has been linked to lymph node metastasis of HCC [20]. We next examined the effect of MMP-13 on HCC cell invasion. Augmented MMP-13 in HepG2 cells markedly increased cell invasion *in vitro* (Figure 3A) and metastasis in nude mice (Figure 3B). In contrast, knockdown of the endogenous MMP-13 using MMP-13 specific shRNAs (shMMP-13) decreased the invasion of HCCLM3 cells *in vitro* (Figure 3C) and metastasis in nude mice (Figure 3D). Notably, MMP-13 increased GP73 expression in HepG2 cells (Figure 3A). On the other hand, knockdown of endogenous MMP-13 decreased GP73 levels in HCCLM3 (Figure 3C). Therefore, MMP-13 also influences the expression of GP73.

### GP73 promotes cell invasion through upregulation of MMP-13 expression

**Figure 4:**
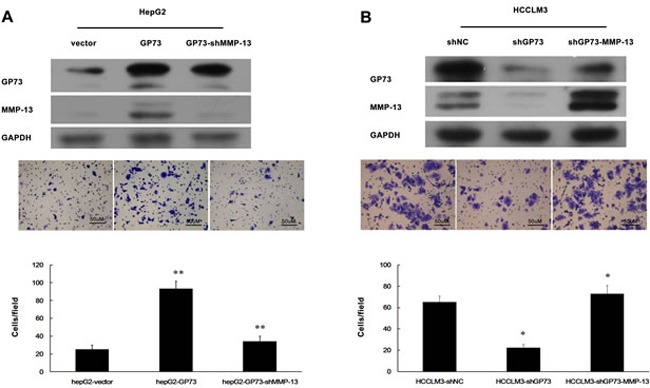
GP73 promotes HCC cell invasion through upregulation of MMP-13 expression Cell invasion was assessed by Matrigel Transwell assay. **A.** MMP-13 was knocked down in HepG2 cells with ectopic GP73 expression. **B.** HCCLM3 cells were first depleted for GP73 and then overexpressed for MMP-13. Western blot (top), transferred cells (magnification, 200×) (middle) and the histograms of transferred cells from triplicate tests (mean ±SD) (bottom).*P < 0.05; **P < 0.01.

**Figure 5:**
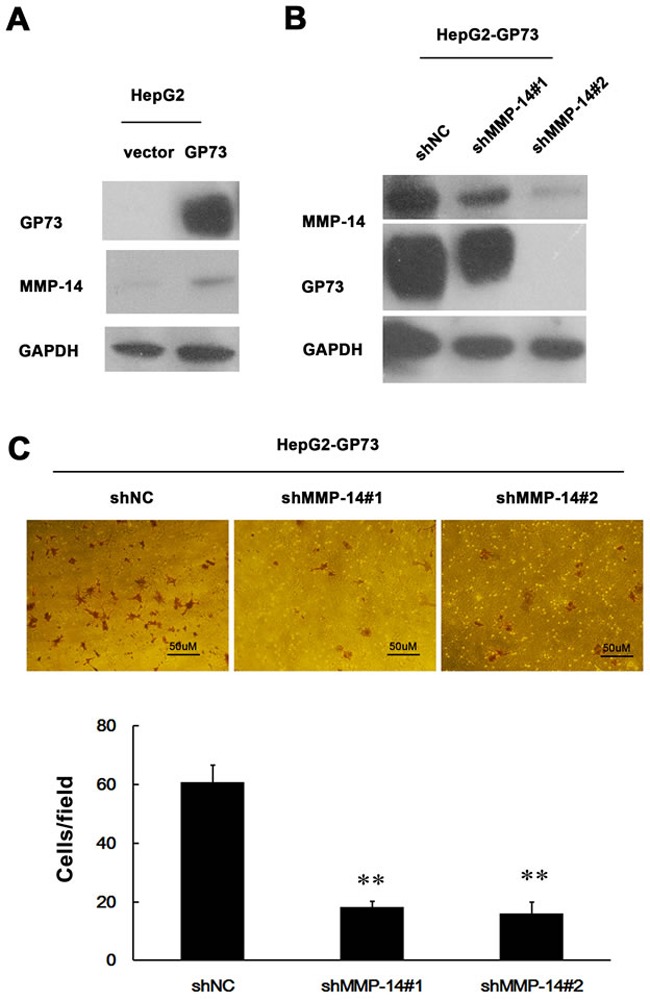
MMP-14 is an effector of GP73 enhanced invasion of HCC cells Western blot analysis for GP73 and MMP-14 in ectopic GP73 expressing HepG2 cells before **A.** and after MMP-14 depletion by 2 interfering RNAs **B.**. **C.** Cell invasion was assessed by Matrigel Transwell assay (magnification, 200×) (top) and the histograms of transferred cells from triplicate tests (bottom) (mean ±SD). **P < 0.01.

Since GP73 enhances MMP-13 expression, we anticipated that GP73 should potentiate cell invasion through MMP-13. Elevated GP73 indeed increased the invasion of HepG2 cells (Figure 4A), while diminished GP73 decreased the invasion of HCCLM3 cells (Figure 4B). Knockdown of MMP-13 abolished GP73 enhanced invasion in GP73-overexpressing HepG2 cells and forced expression of MMP-13 restored invasion in GP73 knocking down HCCLM3 cells (Figure 4A, 4B). Similarly, GP73 also enhanced MMP-14 expression (Figure 5A). Knockdown of MMP-14 reduced GP73 level and compromised GP73 enhanced invasion in GP73-overexpressing HepG2 cells (Figure 5B, 5C). Therefore, GP73 promotes cell invasion by up-regulating MMP-13 and MMP-14 expression.

**Figure 6:**
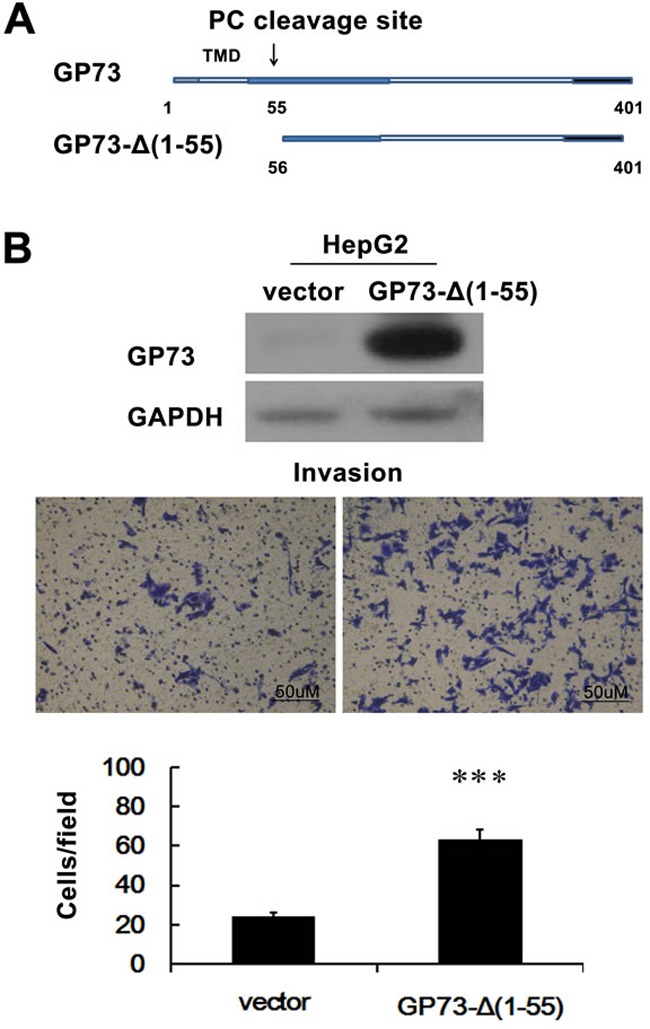
Non-secreted GP73 enhances invasion of HCC cells **A.** Schematic illustration of full-length and mutant GP73. The positions of amino acids are indicated. **B.** HepG2 cells were stably transfected with GP73-Δ(1-55) or PCDNA6 (HepG2-Vector; control). Cell invasion was assessed by Matrigel Transwell assay. ***P < 0.001.

Even though GP73 is an integral Golgi membrane protein, it is also a secreted protein. The N-terminal 1-55 amino acids of GP73 encompass the N-terminal cytoplasmic domain, transmembrane domain, and a PC recognition site, which are crucial for protein Golgi localization and secretion. To test the direct effect of steady-state localization of GP73 on the invasive properties of hepatocellular carcinoma cells, we prepared HepG2 cells that expressed a non-secreted GP73 by transfecting a GP73-Δ(1–55) cDNA, which is devoid of the nucleic acids that encode for the N-terminal 1-55 amino acids needed for secretion [12, 19, 20]. We found that non-secreted GP73 potentiated HepG2 cell invasion (Figure 6).

### GP73 increases HCC cell invasion via activation of CREB-MMP-13-signaling pathway

**Figure 7:**
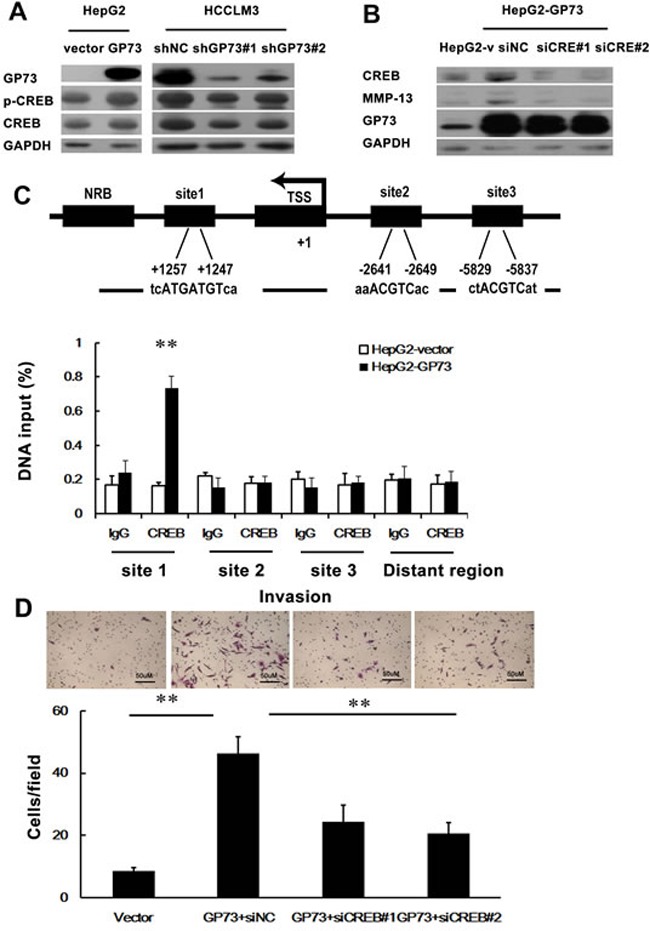
GP73-mediated CREB transactivation of MMP-13 expression The influence of GP73 on CREB expression was demonstrated by Western blot. **B.** The impact of CREB on MMP-13 was illustrated by Western blot. **C.** Schematic representation of the promoter region of human MMP-13 gene. Site1, site2 and site3 indicate the predicted binding region (PBR) of CREB on MMP-13 promoter. PBR and nonspecific binding region (NBR) were amplified in ChIP qRT-PCR analysis. The transcription start site (TSS) is indicated by an arrow above the gene. ChIP assay demonstrated the interaction of CREB with one of the potential CREB-binding sites in MMP-13 promoter. qRT-PCR was performed to detect the amounts of immunoprecipitated products. **D.** The enhanced cell invasion by ectopic expression of GP73 in HepG2 cells was abrogated by depletion of CREB. **P < 0.01.

CREB (cAMP responsive element binding protein) is a nuclear transcription factor that regulates the genes involved in cell survival and cell death. It has been reported that CREB promotes the expression of MMP-13 in human articular chondrocytes and osteoarthritis [21]. We hypothesized that GP73 activates MMP-13 expression via upregulation of CREB. Both total and phosphorylated CREB were increased in the cells with GP73 overexpression, while knockdown of GP73 led to a drastic reduction of CREB expression in HCCLM3 cells (Figure 7A). In addition, knockdown of CREB led to a drastic reduction of MMP-13 expression in HepG2-GP73 cells (Figure 7B), supporting the idea that GP73 increases MMP-13 expression through CREB. We thereby tested whether CREB stimulated MMP-13 expression as a transcriptional activator. We used qRT-PCR analysis of ChIP DNA and found that binding of CREB to a DNA region immediately upstream of the exon 1 of the MMP-13 gene was higher in HepG2-GP73 cells compared to binding in HepG2-Vector cells (Figure 7C). Silencing the expression of CREB partially abrogated the invasive effect of GP73 on HepG2 cells (Figure 7D). These results demonstrate that GP73 promotes HCC invasion through activation of the CREB-MMP-13 signaling pathway.

## DISCUSSION

GP73 is a promising serum biomarker for HCC [4, 22]. However, its role in HCC progression was unclear. Here, we suggest that GP73 promotes invasion of hepatocellular carcinoma cells. This promoting effect of GP73 on cell invasion is achieved through transactivation of MMP-13 expression by CREB. The abundance of GP73 and MMP-13 is positively correlated and enhanced in human HCC tissues.

Cell invasion is the major feature of malignancy. The Golgi apparatus plays an important role in cell migration and acts as a hub for different signaling pathways [23, 24]. Therefore, elucidation of the regulatory networks of the Golgi apparatus will provide important information for the development of therapeutic strategies against cancer migration, invasion and metastasis. GP73 is a Golgi-associated protein and is overexpressed in HCC. We found that overexpression of GP73 promoted the invasion of HCC cells. These data suggest that augmented GP73 functions as an oncogene.

MMPs play important roles in tumor invasion because their proteolytic activities assist in degradation of the extracellular matrix and basement membrane. MMP-13 is expressed in a very restricted manner in the human body, but is often upregulated under pathological conditions, such as cancer and arthritis [25]. It was reported that MMP-13 is upregulated in human HCC [26], however its role in HCC is unclear. We present here that MMP-13 potentiates human HCC cell lung metastasis in nude mice. GP73 accelerates cell invasion through activation of MMP-13 expression. MMP-13 also potentiates GP73 expression. It appears that there is a feedback loop between GP73 and MMP-13. Aberrant MMP-13 expression strongly associates with GP73 abundance in human HCC tissues. The levels of MMP-13 and GP73 are significantly higher in tumors than in their adjacent tissues, therefore, GP73 enhancement of MMP-13 expression may be implicated in HCC development.

Many transcription factors, such as CREB, STAT1, AP-1, and FOXO3a, may regulate the expression of MMP-13 [21, 27]. CREB is one of the best understood phosphorylation-dependent transcription factors that has been shown to regulate the expression of many genes, such as MMPs [25]. In this study, we show that GP73 stimulates the expression CREB and MMP-13. CREB binds to the promoter of MMP-13 and the enhanced CREB is critical for GP73 mediated MMP-13 overexpression. Thus CREB serves as a bridge between GP73 and the MMP-13 signaling cascade and is essential in GP73-MMP-13 mediated cell invasion.

In summary, we demonstrate that GP73 promotes cell invasion by potentiating CREB-MMP-13 expression. Augmented MMP-13 potentiates HCC cell metastasis. We suggest that GP73 enhancement of MMP-13 expression may have clinical relevance in HCC development. Therefore, inhibition of this GP73-CREB-MMP-13 signaling axis should provide a potential therapeutic strategy for HCC.

## MATERIALS AND METHODS

### Cell lines and reagents

Human HCC cell line HepG2 was obtained from ATCC, Bel-7402 cell line was obtained from Peking Union Medical College Cell Culture Center, HCCLM3 and MHCC97L cell lines were from Liver Cancer Institute of Fudan University (Shanghai, China). All cells were cultured in DMEM medium supplemented with 10% fetal bovine serum (FBS, Gibco, Australia), penicillin (100 U/ml), and streptomycin (50 μg/ml) in incubator with humidified atmosphere of 5% CO_2_ and 95% air at 37°C. Antibodies against to human GP73 and MMP-13 were obtained from Abcam (Cambridge, MA, USA). Rabbit anti-CREB/pCREB (Serine 133) (87G3) and anti-GAPDH antibodies were purchased from Cell Signaling Technology (Beverly, MA, USA). The qRT-PCR primers for MMP-1, MMP-2, MMP-7, MMP-9, MMP-13, MMP-14 and glyceraldehyde-3-phosphate dehydrogenase (GAPDH) were synthesized commercially. HRP-conjugated anti-rabbit and anti-mouse antibodies were purchased from Santa Cruz Biotechnology (Santa Cruz, CA, USA). Enhanced chemiluminescence (ECL) system was obtained from Pierce (Rockford, IL, USA). Blasticidin was obtained from Sigma-Aldrich (St. Louis, MO, USA).

### Generation of stable hepG2 cell lines expressing ectopic GP73 and MMP-13

The full-length GP73 and non-secreted GP73 cDNA (GP73-Δ(1-55)) was amplified by forward (5′ CCCAAGCTTGGGATGGGCTTGGGAAACGGG 3′) and reverse (5′ TGCTCT AGAGCAGAGTGTATGATTCCGCT 3′) oligonucleotides, and forward (5′ CCCAAGCTTGGGTTCAACTACTGGATTGC 3′) and reverse (5′ TGCTCTAGAGCAGAGTGTATGATTCCGCT 3′) oligonucleotides, respectively, using a cDNA template obtained from Sino Biological (Beijing, China). MMP-13 cDNAs were amplified by forward (5′ CCCAAGCTTGGGATGCATCCAGGGGTCC TGGCTGCCTTCCTCTTCTT 3′) and reverse (5′ TGCTCTAGAGCATTAACACCACAAAATGGAATTTG CTGGCATGACGCG 3′) oligonucleotides, using a cDNA template obtained from Sino Biological. PCR products were digested with HindIII and XbaI and inserted into a modified pcDNA6/V5-HisB plasmid (Invitrogen, Carlsbad, CA, USA), which contains an V5 epitope at the C-terminus. The plasmids were transfected into HepG2 cells using Lipofectamine 2000 (Invitrogen, Carlsbad, CA, USA). Stable cells were selected using culture media containing 15μg/mL blasticidin for 20-30 days. Stable transformants were maintained in DMEM media containing 10μg/mL blasticidin.

### RNA interference (RNAi)

#### (I) Construction of shRNA plasmid vectors

The sequences targeting human GP73 for RNA interference were (5′ GCAACTCCTAGTAGTACAA 3′) and (5′ GCCAGTGCATCAATCAGATGA 3′). The sequence targeting human MMP-13 for RNA interference was (5′ GACTCATTCTGAAGTTGAA 3′). The sequences targeting human MMP-14 for RNA interference were (5′ GGGTCTCAAATGGCAACATAA 3′) and (5′ GGGAGATGTTTGTCTTCAAGG 3′). Sequence (5′ GTTCTCCGAACGTGTCACGT 3′) targeting none of the known genes was used as negative control. Oligonucleotides of shRNA including a TTCAAGAGA loop motif were synthesized and inserted into pGPU6/GFP/Neo vector with a standard cloning procedure by GenePharma (Shanghai, China).

#### (II) RNA interference with siRNA

The CREB and negative control small interfering RNAs (siRNAs) were purchased from Dharmacon (Thermo Fisher Scientific, Pittsburgh, PA, USA). Cells were plated at a density of 8×10^4^ cells/well in 6-well plates and transfected with 50 or 100 nM CREB or negative control siRNA oligoduplexes after preincubation for 20 minutes with DharmaFect transfection reagent in serum-free Opti-MEM I medium (Invitrogen, Carlsbad, CA, USA). The transfection mixture was replaced with DMEM medium containing 10% FBS (without antibiotics) 5 hours later. Forty-eight hours after transfection, cells were collected for invasion assays or for preparation of whole cell lysates.

### Enzyme linked immunosorbent assay

HCCLM3 and HepG2 cells were transfected with the indicated plasmids for 48 hours. The levels of secreted MMP-13 (active form) in the culture supernatants were measured using MMP-13 Human enzyme linked immunosorbent assay (ELISA) Kit (Abcam, Cambridge, MA, USA) following the manufacturer's instructions.

### RNA extraction, cDNA synthesis, and qRT-PCR

Total RNA was extracted from cell pellets using E.Z.N.A.® Total RNA Kit I (Omega Bio-Tek, Norcross, GA, USA) and reversely transcribed into cDNA using PrimeScript™ RT-PCR Kit (Takara Biotechnology, Dalian, China) according to the manufacturer's instructions. The target gene expressions were determined by qRT-PCR using specific primers of target genes (Table 2). GAPDH was served as an internal control for total cDNA content. For qRT-PCR analysis, aliquots of double-stranded cDNA were amplified using a SYBR Green PCR Kit (TransGen Biotech, Beijing, China). The cycling parameters were 45 cycles of 95°C for 15 seconds, 57°C for 15 seconds, and 72°C for 15 seconds. A melting curve analysis was then performed. The Ct was measured during the exponential amplification phase, and the amplification plots were analyzed using CFX Manager 2.1 software (Bio-Rad Laboratories, Hercules, CA, USA). The relative expression level (defined as fold change) of the target gene was determined by the following equation: 2^−ΔΔCt^ (ΔCt = ΔCt^target^ – ΔCt^GAPDH^; ΔΔCt = ΔCt^expressing vector^ – ΔCt^control vector^). The expression level was normalized to the fold change detected in the corresponding control cells, which was defined as 1.0.

**Table 2:** Primer sequences for real-time PCR sequence

Gene	Forward primer (5′ to 3′)	Reverse primer (5′ to 3′)
mRNA primers MMP-1	CTGGCCACAACTGCCAAATG	CTGTCCCTGAACAGCCCAGTACTTA
MMP-2	TCTCCTGACATTGACCTTGGC	GATCTACTCAGCCAGCACCCT
MMP-3	ACCAACCTATTCCTGGTTGCTGCT	ATGGAAACGGGACAAGTCTGTGGA
MMP-7	TGGACGGATGGTAGCAGTCT	TCTCCATTTCCATAGGTTGGAT
MMP-9	TGACAGCGACAAGAAGTG	CAGTGAAGCGGTACATAGG
MMP-13	CTTCCCAACCGTATTGATGC	TTTGGAAGACCCAGTTCAGA
MMP-14	TACTTCCCAGGCCCCAAC	GCCACCAGGAAGATGTCATT
GAPDH	CATGAGAAGTATGACAACAGCCT	AGTCCTTCCACGATACCAAAGT
CHIP primers		
Binding site 1	ATGTCAGCAATGCCATCGT	CTCATTCTGAAGTCGAAAAGGCAT
Binding site 2	TCAGACATTAGCAATATGGGACTT	TGGCCAATTGTTCATGTTATTCC
Binding site 3	CAGATATTCAGGGGGCTGAG	AATCGGCCTTGTTCTTCTTG
No-binding control	CAGGTGAGGGAAGTGGACAG	AGTGCATCCTCCATCCTTGG

### Cell invasion assay

Cell invasion was investigated using Matrigel-coated 8.0-μm filter invasion chambers (BD Biosciences, San Jose, CA, USA). Cells were incubated for 22 hours at 37°C in a humidified atmosphere of 5% CO_2_. Cells on the upper surface of the membrane were removed using cotton tips after the indicated incubation times. The migrant cells attached to the lower surface were stained with crystal violet (500μL of 5 mg/mL crystal violet dissolved in 20% methanol) and incubated for 30 minutes. The membrane was washed with phosphate-buffered saline, and the cells on the filter were counted under an optical microscope (magnification, 200×).

### Metastasis assays

The pulmonary metastasis of tumors was studied using an intravenous injection model. Nude mice were injected with 2×10^6^ cells in 0.2-mL Hank's solution via the lateral tail vein. After 70 days, mice were euthanized and the lungs were immediately removed and taken photographs for counting of pulmonary metastatic nodules in each of the five lobes. The metastatic tissues were analyzed with H&E staining.

### Chromatin immunoprecipitation assay

Chromatin immunoprecipitation (ChIP) analysis was performed using EZ CHIP kit (Millipore, Billerica, MA, USA). HepG2 cells were grown on 15-cm plates until they reached 85% confluence. Then the cells were fixed and collected for ChIP assay. DNA-protein complexes were precipitated using specific antibody of CREB (Millipore, Billerica, MA, USA). DNA fragments were decross-linked and purified from complexes. Immunoprecipitated and input DNA were used as templates for real-time ChIP-PCR using Sybrgreen Mastermix (TransGen Biotech, Beijing, China). The enrichment of specific DNA sequences was calculated by using the comparative *CT* method [28]. PCR primer sequences are listed in Table 2. All data presented are the results of at least 3 independent ChIP experiments and triplicate qRT-PCR reactions. The ChIP levels for each region are presented as the percentage of input chromatin.

### Human liver cancer analysis

Hepatocellular carcinoma samples were freshly obtained from the patients undergoing surgery at State Key Laboratory of Molecular Oncology, Cancer Institute/Hospital, Chinese Academy of Medical Sciences, Beijing, China. The institutional review board at Cancer Institute/Hospital approved the study protocol, and all patients provided written informed consent. Tumor tissues were snap frozen and later were sonicated for immunoblotting [29, 30].

### Statistical analysis

The values were reported as means ± standard error of the mean (SEM). The statistical significance of differences between the means of 2 groups was evaluated by paired Student's *t*-test. In all cases, *P* < 0.05 was considered significant. Statistical analysis was performed using Statistical Package for Social Sciences (SPSS) software (version 16.0).

## Abbreviations

GP73: Golgi protein 73
MMP-13: matrix metalloproteinase-13
CREB: cAMP response elementbinding protein
HCC: human hepatocellular carcinoma
ELISA: enzyme-linked immunosorbent assay
GAPDH: glyceraldehyde-3-phosphate dehydrogenase
